# Corrigendum to “miR-375 Inhibits the Proliferation and Invasion of Nasopharyngeal Carcinoma Cells by Suppressing PDK1”

**DOI:** 10.1155/2020/3595402

**Published:** 2020-10-30

**Authors:** Xu Jia-yuan, Song Wei, Lu Fang-fang, Dai Zhi-jian, Cao Long-he, Lin Sen

**Affiliations:** Department of Otolaryngology, The Third Affiliated Hospital of Wenzhou Medical University, Ruian, Zhejiang, China

In the article titled “miR-375 Inhibits the Proliferation and Invasion of Nasopharyngeal Carcinoma Cells by Suppressing PDK1” [1], the *Y*-axes labelling of Figures 1(a) and 1(b) were inverted by mistake. The corrected figure is shown below and is listed as Figure 1.

## Figures and Tables

**Figure 1:**
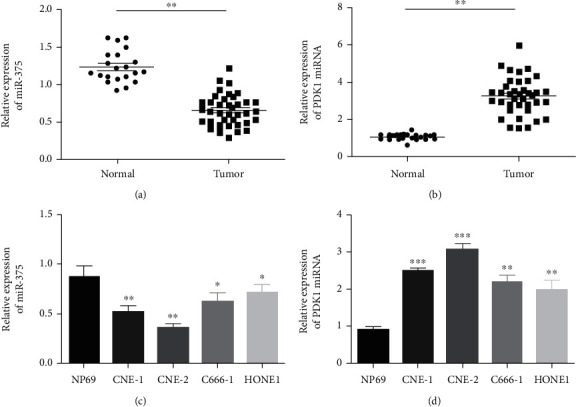
Expression levels of miR-375 and PDK1 in NPC tissues and cell lines were assessed. (a, b) qRT-PCR was performed to measure expression levels of miRNAs and PDK1 in 23 normal samples and in 38 NPC samples. miR-375 is downregulated in NPC tissue, compared with normal tissue, while PDK1 is upregulated in NPC tissue, compared with normal tissue (Student's *t*-test, ^∗∗^*P* < 0.01). (c, d) qRT-PCR was performed to evaluate miR-375 expression in the N69 epithelial normal nasopharynx cell line and NPC cell lines (CNE-1,CNE-2, C666-1, and HONE1). Conversely, levels of PDK1 were markedly increased in NPC cell lines, compared to NP69 (CNE-1, CNE-2, C666-1, and HONE1) (Student's *t*-test, ^∗^*P* < 0.05, ^∗∗^*P* < 0.01, and ^∗∗∗^*P* < 0.001). Cell lines CNE-2 and CNE-1 showed the greatest difference in miR-375 expression levels when compared to NP69 cells and were used for subsequent analyses. Each assay was conducted in duplicate, three times. Values are presented ±s.e.m.
